# Proprioception Is Robust under External Forces

**DOI:** 10.1371/journal.pone.0074236

**Published:** 2013-09-03

**Authors:** Irene A. Kuling, Eli Brenner, Jeroen B. J. Smeets

**Affiliations:** MOVE Research Institute Amsterdam, Faculty of Human Movement Sciences, VU University, Amsterdam, The Netherlands; University of Reading, United Kingdom

## Abstract

Information from cutaneous, muscle and joint receptors is combined with efferent information to create a reliable percept of the configuration of our body (proprioception). We exposed the hand to several horizontal force fields to examine whether external forces influence this percept. In an end-point task subjects reached visually presented positions with their unseen hand. In a vector reproduction task, subjects had to judge a distance and direction visually and reproduce the corresponding vector by moving the unseen hand. We found systematic individual errors in the reproduction of the end-points and vectors, but these errors did not vary systematically with the force fields. This suggests that human proprioception accounts for external forces applied to the hand when sensing the position of the hand in the horizontal plane.

## Introduction

When making a goal directed movement, information from both vision and proprioception is used to identify the position and orientation of the hand [1–7]. With this information, people are able to reach for an object and use it; for example to grasp a cup of coffee and bring it to their mouth to drink. Despite the ease with which we perform such tasks, there is evidence that subjects have considerable biases when matching proprioception to vision [8–11]. These biases suggest that the calibration of our senses is far from perfect. It is hard to say whether this mismatch in calibration is due to errors in vision, proprioception or both. In this study we examine a complication of proprioception that may contribute to such biases: external forces.

Why might proprioception not be perfect? In general, proprioception is based on two types of information that humans can use to know where their limbs are: afferent and efferent information. Efferent information provides an estimate of the intended posture, and could provide information in advance, whereas afferent feedback provides delayed information about the achieved posture, derived from cutaneous, muscle and joint receptors (e.g. [12,13]), that all deliver information about the position of the arm and hand relative to the body.

The use of efferent information can lead to systematic errors when the relation between generated force and position is perturbed. Smith et al. [14] have shown that if subjects are unable to move a finger due to mechanical restrictions or because the forearm and hand muscles are paralyzed, their voluntary effort to move the finger (i.e. efferent information) changes their sense of the position of that finger. Polit & Bizzi’s [15] finding that deafferented monkeys were able to point at a target indicates that efferent information can be reliable enough to perform some tasks as long as the movement is not disturbed. When an external force was applied to the deafferented monkeys’ arms, the monkeys showed a change in movement endpoint that was consistent with not knowing about the external force. Since unknown external forces will bias efferent position sense, relying on efferent information does not seem to be a reliable strategy. However, the combination of this efferent information with afferent information might have considerable advantages that might be more important than the introduction of systematic errors.

An evident reason for considering efferent information, other than circumventing the inevitable delays in afferent information, is that afferent information is also not perfect. The relation between the firing rate of various receptors and the position of the arm is not a simple one-to-one mapping, but depends in two ways on the amount of exerted force. Firstly, the dynamic characteristics of the muscle tendons make the relation between joint angle and muscle fiber length quite complex [16]. For example, when muscles exert more force with the same total muscle length, the muscle tendons are slightly stretched and the muscle fibers, and thus the lengths of the fibers, are a little shorter. Secondly, gamma-activation adjusts the relation between the muscle length and firing rate. To change the position of the arm, an efferent (alpha) signal is sent to the muscle. At the same time another efferent signal (gamma-activation) is sent to the intrafusal muscle fibers that influence the muscle spindles’ state to changes in muscle length [17]. In the absence of a precise and completely reliable source of information, it makes sense to combine all possible information, weighted according to their precision (e.g. [1–3,18]) and possibly also accuracy [19].

There are reasons to believe that external forces will affect the combined sense of position. Debats et al. [20] used a simplistic model of the human arm to explain various experimental results on the radial-tangential illusion by the differences in muscular torque changes between the movements. Their study suggests that both afferent and efferent signals (possibly alpha and gamma-activation) play a role in position sense, and that even ‘natural’ external forces (i.e. gravity) can disturb the sense of position or displacement. From this study and the ones discussed above [14,15], we would expect that an explicit manipulation of external forces would certainly influence the sense of position. In contrast with this expectation, Cordo & Flanders [21] argued that external forces hardly influence the perceived position of the hand. They showed that if a movement is distorted by changing the stiffness of a spring load (0.3-0.5 Nm/deg) on the elbow, people are still able to correctly indicate when their hand is in a defined target zone.

In the present set of experiments we extended the basic idea of Cordo & Flanders’ [21] experiment to a situation with fewer movement restrictions; instead of a spring load on the elbow in a device with a single degree of freedom, we applied external position-dependent force fields to the hand with a force feedback device.

In the first experiment, subjects had to reach visual targets with their unseen hand, forcing them to rely on their proprioceptive position sense. In the second experiment we added trials in which subjects had to reproduce a movement vector instead of moving to an indicated position, requiring the use of proprioceptive sense of displacement. In both the first and the second experiment the external forces depended on the hand’s position in space but not on its movement direction. In the third experiment we used force fields that changed in accordance with the direction of individual movements. We expected for all three experiments that differences between the movements with and without external forces would correspond with the characteristics of the force fields.

## Experiment 1

In the first experiment we examined whether external force fields induce changes in the judged positions of the hand that are related to the direction of the external force field.

### Methods

In this experiment the subject had to reach the positions of targets with the handle of a force feedback device. The targets were distributed in a horizontal plane. A horizontal force field was imposed on the subject’s hand through the force feedback device.

#### Subjects

Twelve subjects (two left-handed, three men, 18-33 years of age) volunteered to take part in the experiment, including one of the authors. Three of the subjects performed experiment 2 before participating in this experiment. All except for the author were naive about the purpose of the experiment. All subjects reported (corrected-to-) normal vision.

#### Ethics Statement

The experiment is part of an ongoing research program that has been approved by the ethics committee of the Faculty of Human Movement Sciences of VU University. All subjects gave their written informed consent.

#### Data availability

All raw data can be requested by sending an email to the corresponding author (i.a.kuling@vu.nl).

#### Stimulus and Apparatus

**Figure 1:**
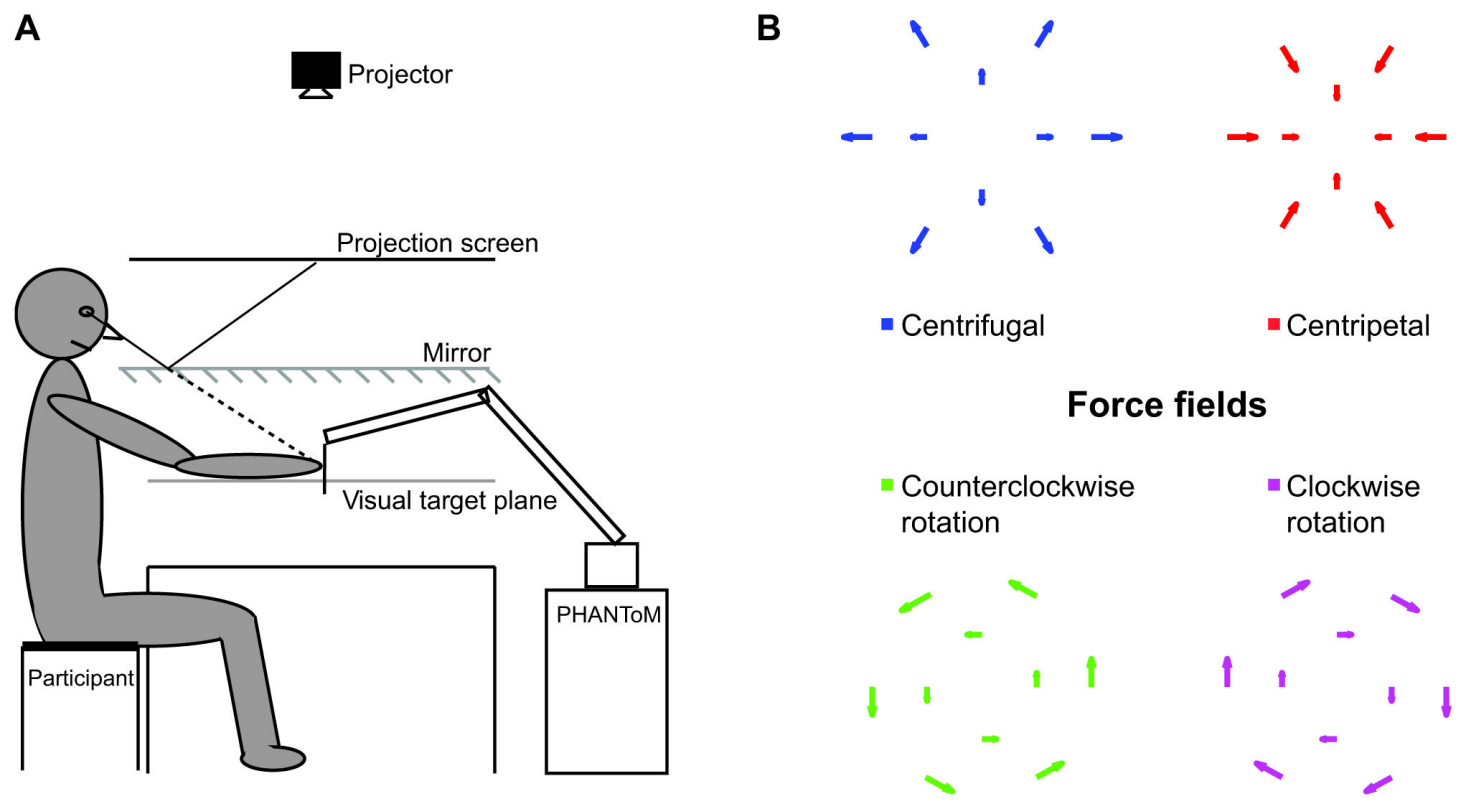
Experimental setup. A) The subject saw the reflection of the target projected on the projection screen. The targets were perceived in a virtual plane between the mirror and the table. B) Top view of the force fields: Blue and red show the centrifugal (CF) and centripetal (CP) force fields. Magenta and green show the clockwise (CW) and counterclockwise (CCW) force fields.

We projected the visual target stimuli on a white see-through projection screen above a mirror. The mirror reflected the images, so that the subjects perceived the targets in a plane below the mirror (Figure 1A). Subjects moved their hand below the mirror, holding a PHANToM Premium 3.0/6DoF (SensAble Technologies) force feedback device, which was used to create the force fields.

The target stimulus was a disc (radius = 1 cm). The color of the disc provided feedback about the height of the handle to prevent the subjects from hitting the mirror. The disc was green as long as the handle was not more than 30 mm above or below the plane of the targets. Above this range the disc turned red, and below this range it turned blue. Subjects were informed about this color-coding and instructed to keep the target green.

Five different force fields could be presented in the workspace: null (without forces), centripetal (CP), centrifugal (CF), clockwise rotation (CW) and counterclockwise rotation (CCW) (Figure 1B). For all force fields, the force was zero at a central point of the workspace (origin), which was aligned with the midline of the subject’s torso and about 30 cm in front of the subject. The forces increased with the distance from the origin by 25 N/m in the horizontal plane, and were independent of the vertical position of the handle.

The visual targets were shown at ten different positions (Figure 1B). Six outer target positions were at a distance of 10 cm from the origin. Four inner targets were at a distance of 5 cm from the origin. At the six outer target positions the magnitudes of forces were equal (because they were at equal distances from the origin); the force on the hand was 2.5 N. At the inner target positions the force on the hand was 1.25 N. These forces were large enough to be clearly felt, but not so large as to make it difficult to move the hand in any desired direction. Note that the actual forces on the hand when matching the visual targets depended on the proprioceptive bias of the subject in question. For example, if a subject had a proprioceptive bias away from the body, the force on the hand when it is perceived to be at the closest targets would be smaller than 2.5N, while for the furthest targets the forces would be larger than 2.5N.

#### Procedure

**Figure 2:**
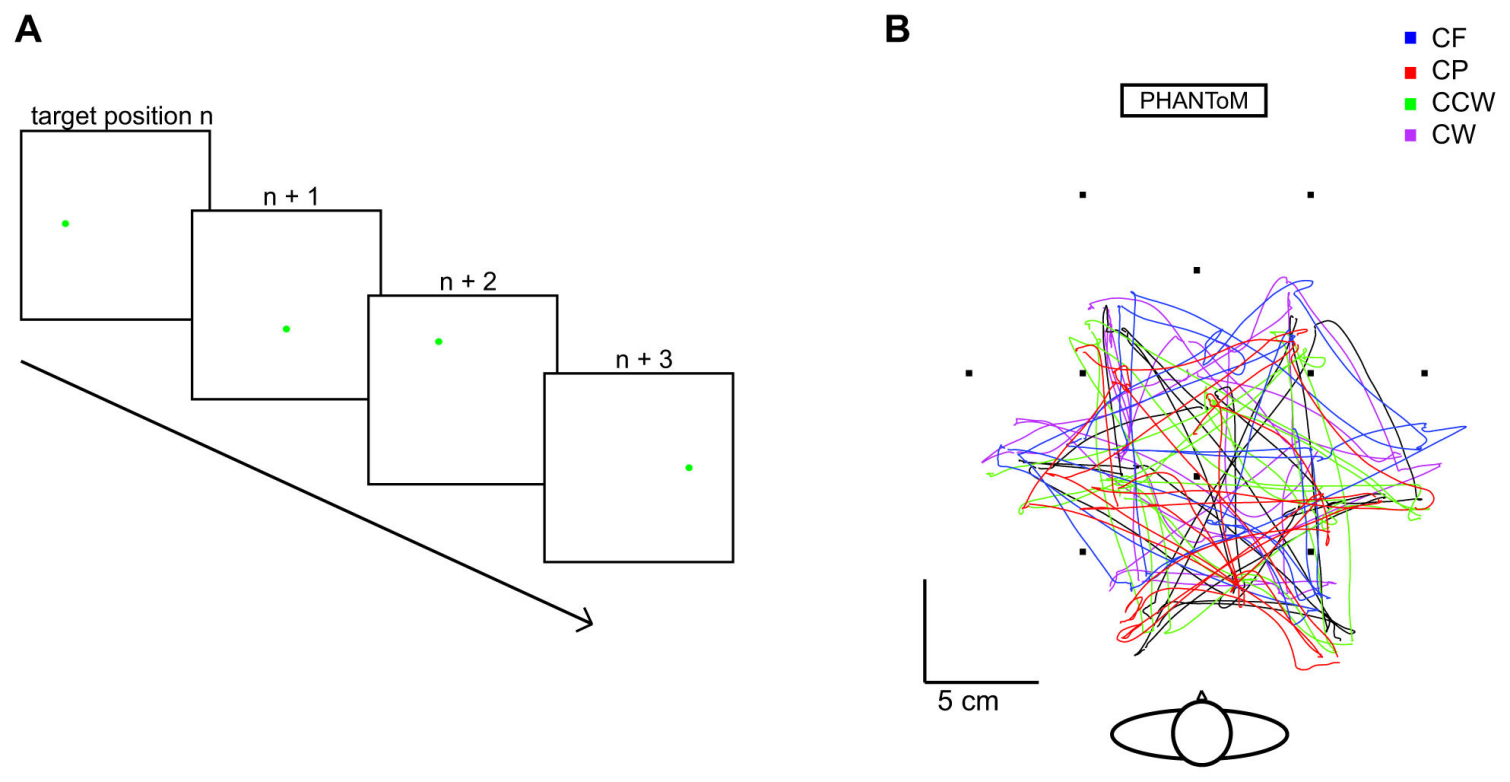
Methods of Experiment 1. A) Sequence of stimuli for four trials in Experiment 1. A disc was shown; the subject moved to that position and pressed the button on the phantom; a new disc appeared, etc. B) Example of a sequence of 20 movement paths of subject 1 in all 5 force fields. Color-coding as in Figure 1B.

The subjects received verbal instructions about the task. They had to hold the handle of the PHANToM force feedback device in their right hand and move their hand together with this handle to the position at which they perceived the target. When they were satisfied that the handle was aligned with the visual target, they pressed the button on the PHANToM and the next target appeared (Figure 2A). Each trial started at the position where the previous trial ended; each target position was thus reached from up to nine different starting positions. Subjects did not receive any feedback during the experiment other than from their own proprioception and the possible warning color about the height of the hand. The position of the subject’s hand was tracked by the PHANToM during the whole experiment.

Each force field was presented to every subject as a block of 162 trials in which each of the ten target positions was presented 16 times. The first and the last (162nd) target of each block were located at the origin to avoid a sudden onset or loss of force on the subject’s hand. The remaining 160 trials were presented in 16 consecutive sequences, each containing the 10 target positions in a semi-random order (not completely random because the first target of a sequence could not be identical to the last one of the previous sequence).

The force fields were presented in a counterbalanced order across subjects. A block of trials (one force field) took the subjects 4-7 minutes. After each block there was a break of 3-5 minutes. On average it took the subjects 45 minutes to complete the whole experiment.

#### Analysis

First, for each subject and force field, the mean of the end-points of the 16 movements to each of the 10 target positions was calculated and compared with the positions of the visually presented target. This revealed the individual patterns of errors and gave a first impression of the differences and similarities between alignments for the different force fields. Next, we calculated *error fields*: the difference in position error between blocks with a force field and the block with the null field.

We anticipated that the force fields would influence proprioception in a way that is proportional to the forces, so the end-points were analyzed by fitting *model fields* to the data. We used three different model fields: two of them (expansion and rotation) were proportional to the forces in one of the four force fields in Figure 1b. The third model field was a translation of the complete pattern of end-points. We expected a rotational model field to fit the error fields for the CW and CCW force fields best, and an expansion field to fit the error fields for the CF and CP force fields best. We examined the extent to which each of the model fields could explain the error fields. The fits of the rotation field and expansion field were both one-parameter fits; the parameters being the angle of rotation and the expansion factor, respectively. The origin of the fitted rotation and expansion was fixed at the actual origin (in accordance with the force fields). Both fits were performed on the errors for all force fields to allow us to compare the fits of models that are related to the force fields with ones that are not. To further check for systematic effects of the force fields that do not match the characteristics of the force fields we also fit a translational model field (a uniform shift of the endpoints) to all the error fields. This fit had two parameters (direction and extent).

Although we were primarily interested in the hand positions that were judged to match the visual targets, we also examined the force fields’ influences on the movements themselves. To test whether the forces affect the movement paths, we determined the length of the path travelled (‘actual path’, Figure 2B) and the length of the vector between the start and end of the movements (‘shortest path’) for each movement. The ratio of ‘actual path’ length to ‘shortest path’ length is a measure of how straight the paths were. We will refer to this measure as *extra path*. This ratio was averaged across trials for each subject and force field.

### Results

**Figure 3:**
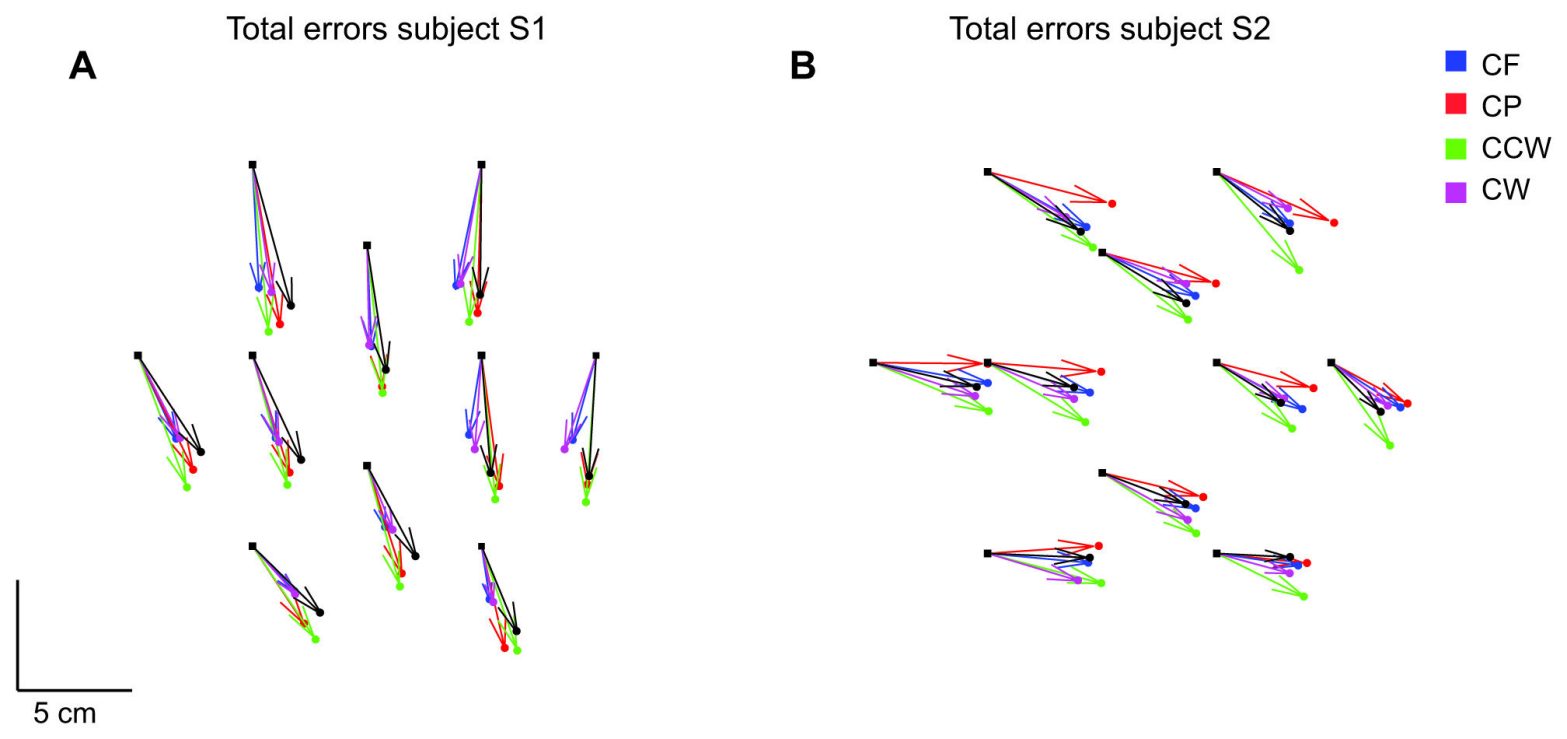
Example results of Experiment 1. The end-point errors for two subjects. The arrows connect the presented positions (squares) with the mean of the indicated end-points. Color-coding is the same as in Figure 1B.

The data of one of the subjects in experiment 1 were excluded from the analysis, because of an experimental error in the null force field. For the other eleven subjects, the mean end-point positions were calculated and compared with the visually presented target positions. For all subjects, the number of target color changes due to changes in the hand’s height was low (about 8 per block of trials), so vertical displacements were not analyzed. The two subjects whose data are presented in Figure 3 show comparable errors for all force fields. The black squares show the visual target positions and the dots are the matching positions for each force field. The arrows show the errors for each force field relative to the visually presented target positions. The errors are comparable for all force fields, but the pattern of errors differed considerably between the two subjects, which is consistent with the subject-specific patterns of errors found in other experiments [8–11].

**Figure 4:**
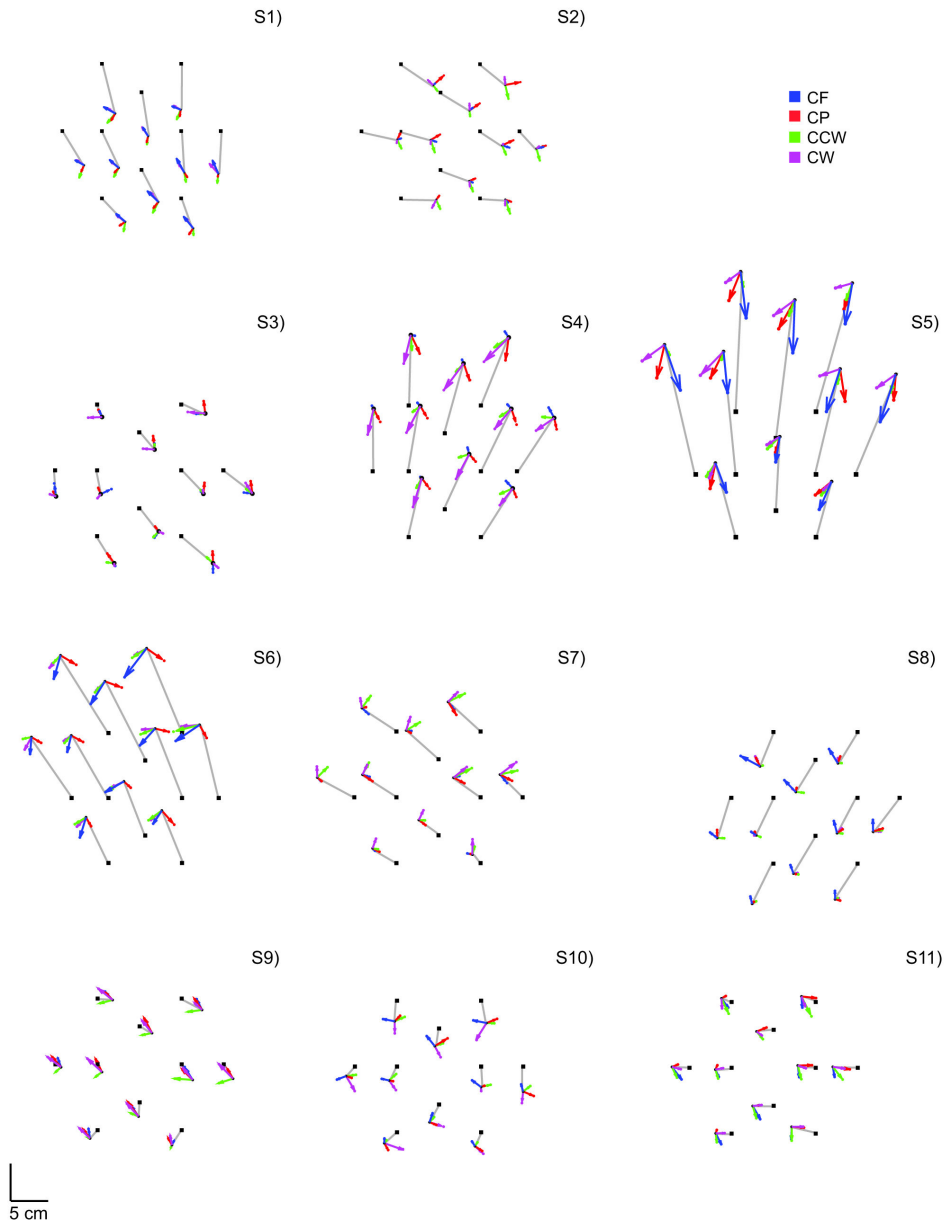
Error fields of all subjects of Experiment 1. Each graph shows the error fields (differences between the errors in the null force field and in the other force fields) for one subject. The thin lines indicate the errors in the null force field. Magenta and green arrows show the errors in the clockwise and counterclockwise rotation force fields relative to the null force field. Blue and red arrows show errors in centrifugal and centripetal force fields relative to the null force field. The differences between the force fields are much smaller than the mismatch with the visually presented target positions.

Figure 4 shows the error fields (see section ‘analysis’ for details) for each subject. The arrows show the differences between the reached positions with and without force fields. These differences are generally smaller than the differences between the visually presented and the indicated positions (grey lines).

The force-related rotation and expansion model fields (see ‘analysis’ section for details) were fitted to the error fields. The proportion of unexplained variance was calculated for each fit, providing a measure of the extent to which the transformation accounts for the data. None of the related fits decreased the unexplained variance substantially (i.e. by more than about 10-20%, see Table 1, bold numbers). Although the mean residual unexplained variance was slightly lower after three of the four related fits than after the corresponding unrelated fits, a repeated-measures ANOVA (4x2) (force field x related/unrelated fit) on the proportion of unexplained variance showed no significant difference between the related and the unrelated fits (F_(1,10)_ = 1.03, p=.34). There was also no significant effect of force field (F_(3,30)_ = 1.02, p=.40) nor an interaction between the two factors (F_(3,30)_ = 2.21, p=.11).

**Table 1:** The fraction of unexplained variance (means over subjects) for all combinations of force field and model field in Experiment 1.

Force field	Model fields		
	Rotation	Expansion	Translation
Centrifugal	0.91	***0.81***	0.32
Centripetal	0.97	***0.79***	0.25
Counterclockwise	***0.90***	0.82	0.21
Clockwise	***0.84***	0.83	0.34

The total variance is the sum of the squared differences between the errors for the null force field and for the other force fields. Bold numbers are combinations in which the model field matches the force field. The fits of the rotation and expansion model field leave most variance unexplained. The translation model field explains slightly more of the variance, but is unrelated to the presented force fields.

The reduction in the amount of unexplained variance by the translation fit (Table 1, last column) showed that there may be some global shifts in proprioceptive judgments for the different force fields. The fits could account for about 70% of the variance. The shifts (ca. 1-2 cm) might be due to random fluctuations in the bias, possibly as a result of slight changes in posture or in the head position relative to the apparatus (and thus the viewing angle) between the blocks.

**Figure 5:**
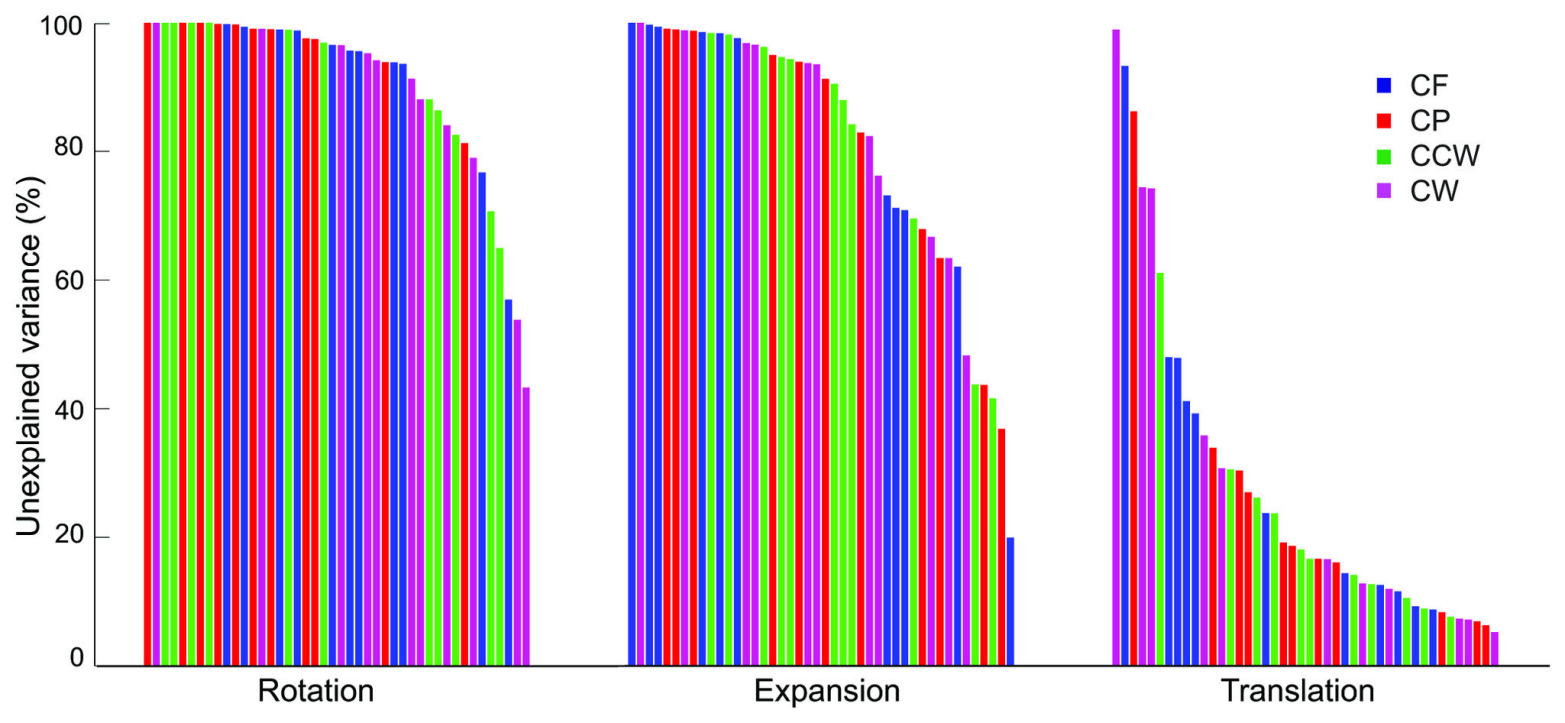
Possible effects of force fields on position matching. The data of each subject-force field combination in Experiment 1 are independently matched with a rotation, expansion and translation model field (the three panels). Each bar within a panel represents one force field of one subject. Color-coding as in Figure 1B. The bars are ordered from high to low unexplained variance.

Another way to show the extent to which the force fields can account for the differences is by plotting the fractions of unexplained variance for all subjects individually in all force field-fit combinations (Figure 5). For each model field the bars are sorted from high to low fractions of unexplained variance. The unexplained variance was reduced most with a translational model field. To test whether there was an overall difference between the effects of related and unrelated model fields, a Mann–Whitney U-Test was done. This test showed no differences in explained variance between the related and unrelated force fields (rotation: U_(22.22)_ = 179, p = 1.48 and expansion: U_(22,22)_ = 223, p = 0.45).

Thus, altogether the results showed that, in contrast with our expectation, the reduction of unexplained variance by the various model fields (rotation, expansion or translation) was unrelated to the characteristics of the force fields (rotation or expansion).

**Figure 6:**
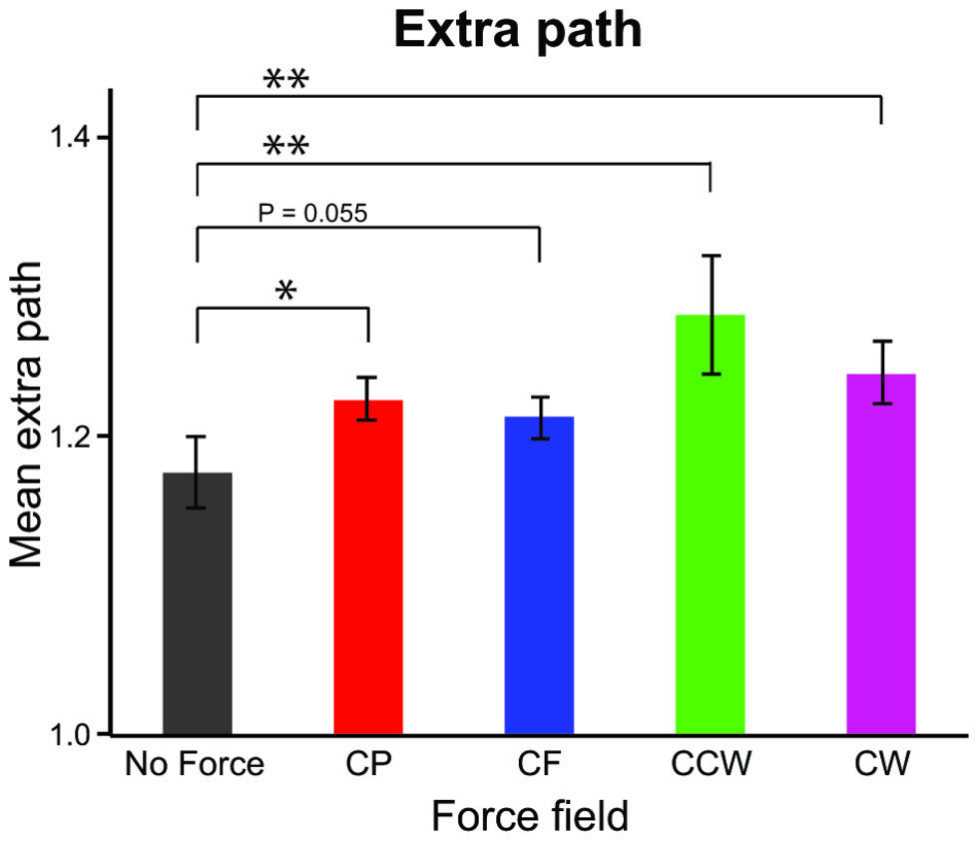
Mean extra path for the five different force fields. Color-coding as in Figure 1B. Each bar shows the mean of the extra path for all subjects in Experiment 1. Error bars show SE’s. The extra path is shorter without forces than with forces. The asterisks denote that the difference is significant (* = p < 0.05, ** = p < 0.01).

The subject’s path from start to end position was on average 1.2 times longer than the shortest distance between start and end position. A repeated–measures ANOVA shows that there was an effect of external force on the amount of extra path (F_(4,40)_ = 5.67, p < 0.01). Post hoc comparisons showed that the extra path was significantly longer in the presence of a force field than when there was none (Figure 6).

### Discussion

In experiment 1, the force fields did not have a systematic effect on subjects’ manual matching of visually presented target positions. Each subject had his own spatial pattern of errors with respect to the presented target positions, but this pattern did not change in the presence of the external force fields. We found that the force fields influenced the amount of extra path, which shows that the forces did have an effect on the movement paths. The forces increased the amount of extra path. Thus, subjects could adequately reach the desired end positions despite moving differently as a result of changes in the forces on the hand, which is in line with the findings of Cordo & Flanders [21]. Unlike results of the paths from position-matching trials in velocity-dependent force fields (e.g. [22]), we did not find an adaptation or learning effect during the task (not shown). This might be because the position-dependent force fields were not related to the hand movement itself and the paths varied across space, which made the force profiles different for each movement.

The fact that we did not find an effect of forces on the end positions, but only on the paths, might be a consequence of the type of motor planning that was required to perform this task. Human arm movements can be seen as an initial vector-based movement combined with (or followed by) end-point control (e.g., [23,24]). An unpredictable force on the hand might influence the vector-based part of the movement more than the end-point control, because the initial vector-based part is likely to rely more on efferent information than the final correction for reaching the end-point. To test this hypothesis we designed a second experiment in which the vector-based component is likely to be more important. In this new task we did not provide the endpoint of the movement, but a distance and a direction that had to be moved.

## Experiment 2

In the second experiment subjects alternated between two tasks; moving to visually presented targets (as in Experiment 1) and reproducing vectors. To reproduce a vector, both the direction and the length (distance travelled) had to be estimated and reproduced. As the forces on the hand are not purely parallel or perpendicular to the movement direction, we anticipated that both the direction and the length of the movement could be influenced by the force fields.

### Methods

#### Subjects

Nine subjects (two left-handed, one man, 24-33 years of age) volunteered to take part in the experiment. Four of the subjects had taken part in Experiment 1 before they participated in this experiment. All were naive about the purpose of the experiment. All subjects reported (corrected-to-) normal vision.

#### Stimulus and Apparatus

The apparatus and the force fields were identical to those in Experiment 1, but the stimuli differed. The main difference (see Table 2) was related to the fact that there were two types of trials in Experiment 2: position matching trials (that were identical to the trials in Experiment 1) and vector reproduction trials. In the vector reproduction trials the stimulus consisted of two items: an arrow (length = 1 cm) at the start position, which indicated the direction of the vector, and a line on the right side of the projection screen, that indicated the length of the vector. Since the visual length information was presented at a fixed position and with a fixed orientation, separately from the visual direction, subjects had to transfer the extent to a different position and orientation. Although transferring the estimate of the required movement amplitude to the indicated position and direction could introduce systematic errors, errors arising from this will be the same for all force fields.

**Table 2:** Overview of the experimental designs of the three experiments.

	**Experiment 1**	**Experiment 2**		**Experiment 3**
**Session**	Single	1	2	Single
**Force fields (FF)** *Blocks in counterbalanced order*	Null	Null	Null	Null
	Centrifugal (CF)	CF	CW	Assisting 1N
	Centripetal (CP)	CP	CCW	Assisting 2N
	CW rotation			Resisting 1N
	CCW rotation			Resisting 1N
**Task**	Position-matching	Position-matching Vector reproduction		Position-matching
**Number of targets**	10 positions	18 position-vector pairs		9 positions
**Repetitions** *Targets in pseudorandom order*	16 per position	8 per position-vector pair		8 per position
**Workspace (lxw)**	20 cm x 20 cm	20 cm x 20 cm		26 cm x 40 cm

**Figure 7:**
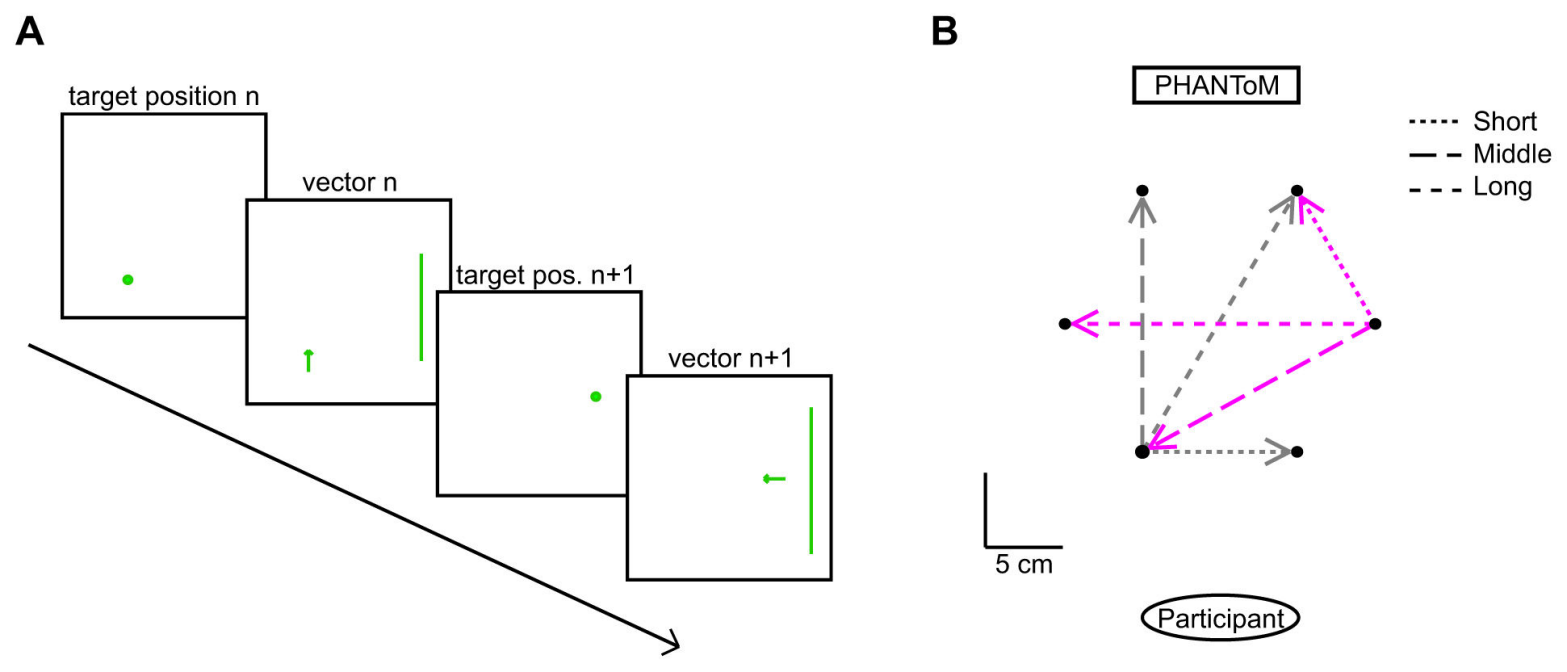
Methods of Experiment 2. A) Sequence of four trials in Experiment 2. First a disc was shown; the subject moved to that position and pressed the button on the PHANToM. Then an arrow and line appeared and the subject had to make a movement in the direction of the arrow over a distance corresponding to the length of the line. This pair of trials was then repeated, but with other positions, directions and line lengths. B) Six of the 18 target vectors (presented as separate distance and direction information, see panel A). There were three possible vectors from each target position (indicated by different dashed lines). All vectors were rotated to the orientation of the three grey vectors for the analyses.

There were six target locations for the position trials, all at a distance of 10 cm from the origin. These corresponded to the outer target locations of Experiment 1, so the force was 2.5N at all target locations. Each trial started at the position at which the previous trial ended, so position trials were included to avoid drift caused by an accumulation of errors in the vector trials. The position trials and vector trials were presented in pairs, so that in the vector trials, the arrow was shown at the target location of the previous position trial (which was the position at which the subject judged his or her hand to be at that moment). Each of the six target locations was combined with three different vectors, giving a total of 18 pairs. The chosen vectors were the vector to the nearest counterclockwise neighboring target location, to the furthest (opposite) target location and to the second-nearest clockwise neighboring target location. An illustration can be seen in Figure 7B.

#### Procedure

Every subject took part in two sessions. Each session consisted of three blocks: a block without forces, and one block each for two of the force fields. Each block had 288 trials, with a break of about 2 minutes after 144 trials. The blocks with centripetal and centrifugal force fields were always presented in one session, and the clockwise and counterclockwise rotation force fields were presented in the other session. The sessions were presented in a counterbalanced order across subjects. Within each block each pair of target position and vector was presented eight times. Pairs of trials were presented in semi-random order as in Experiment 1.

The subjects received verbal instructions about the tasks. They had to hold the handle of the PHANToM force feedback device in their right hand and move their hand to the position at which they perceived the target disc (position task). When they were satisfied with the position, they pressed the button on the PHANToM and vector information appeared. They had to move in the direction of the arrow by the distance that the line length indicated (vector task). When they were satisfied, the subjects had to press the button again, and a new target disc appeared (Figure 7A). Subjects did not receive any feedback during the experiment other than from their own proprioception and the possible warning color about the height of the hand. The subjects were instructed to keep their hand at the same height. The position of the subject’s hand was tracked by the PHANToM during the whole experiment.

It took subjects about 5 minutes to complete a block of trials. After each block there was a break of 2-3 minutes. A session took about 45 minutes. The two sessions were measured on different days within a two-week period.

#### Analysis

The results of the end-point trials were analyzed as in Experiment 1. For the vector trials, the mean distance and direction moved was calculated for each subject, force field and presented vector. The amount of extra path was calculated and compared over force fields and tasks. The mean trial duration was calculated to see whether the different tasks take different amounts of time to complete.

### Results

For the end-point trials of Experiment 2, the results were similar to those in Experiment 1. Each subject had his own specific pattern of errors, but the force fields did not influence the end-points. All subjects, except one, made shorter movements than the presented lengths. The shortest path lengths were about 88% of the presented line lengths.

**Figure 8:**
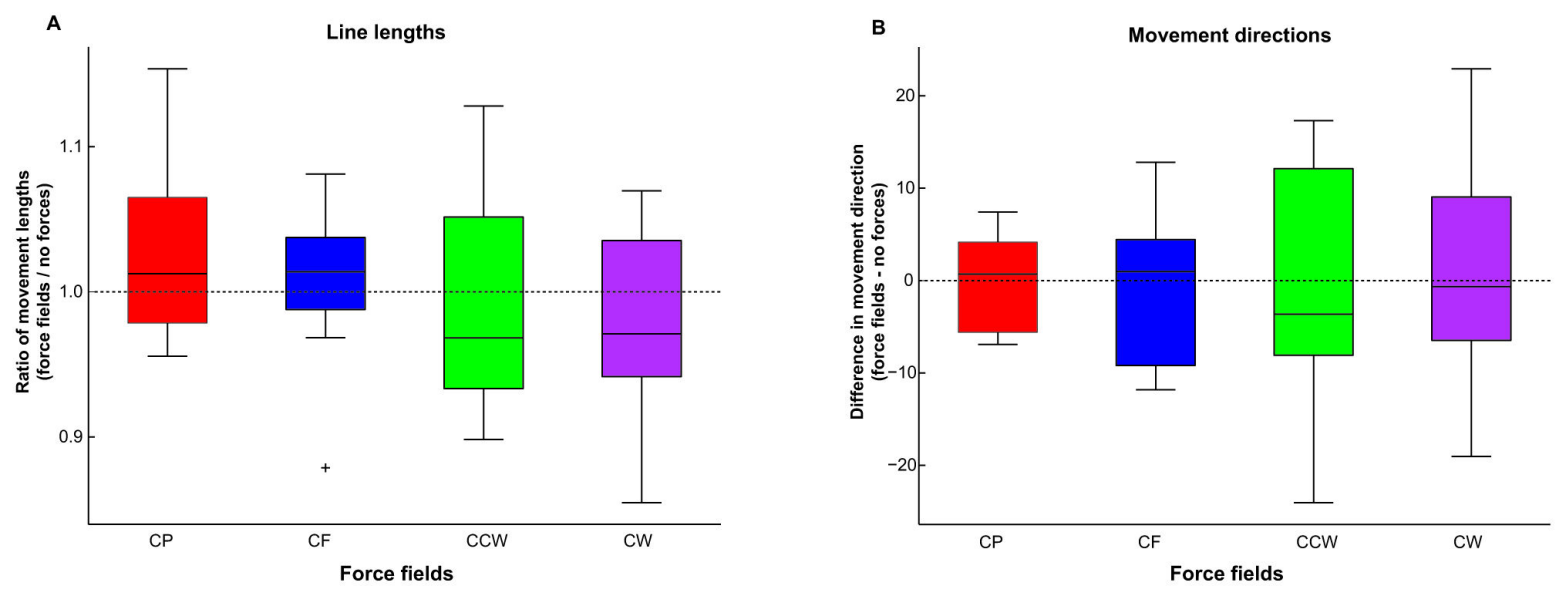
Results of vector trials in Experiment 2. The mean ratio between the distances moved (**A**) with force fields and without forces. B) shows the difference in movement directions with force fields and without forces. Color-coding as in Figure 1B. Both distances and movement direction did not change with the force fields.

In Figure 8A the mean ratios between the distances in the force fields and the null force field are plotted in a boxplot. In Figure 8B the mean differences between the directions of the movements are plotted. *t*-Tests showed that these ratios between the lengths in the force fields and without forces did not differ from one (all |*t*|’s < .34, all p’s > .74). Also for the differences in movement directions *t*-tests showed no significant effect between the directions in the force fields and without forces (all |*t*|’s < 1.23, all p’s > .25). This means that there were no systematic effects of the forces on the distance and direction moved.

**Figure 9:**
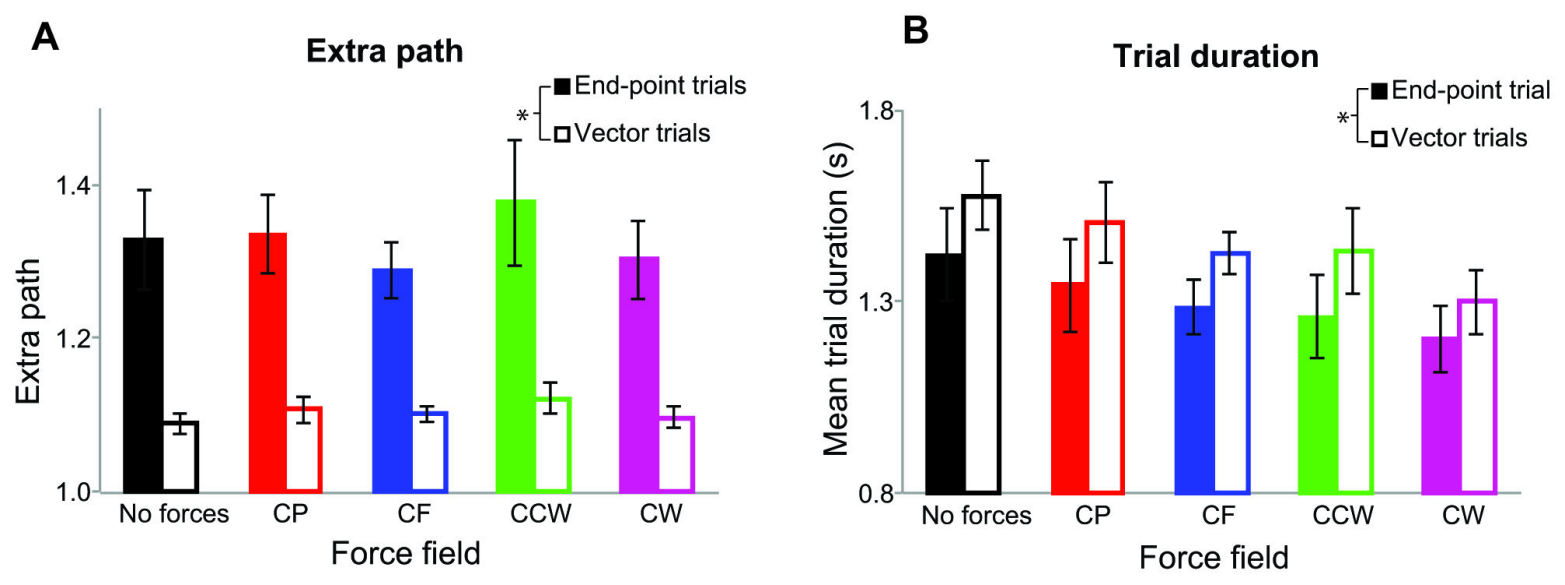
Results of Experiment 2. **A**) Extra path for the five different force fields and two different trial types. Filled bars show the results of the end-point trials and open bars of the vector trials. In the vector trials the extra path is significantly shorter than in the end-point trials (p<0.01). **B**) Mean trial duration for the different force fields and trial types. In the vector trials the mean trial duration is significantly longer than in the end-point trials (p<0.05). Error bars show standard errors.

For the movement paths, a repeated-measures ANOVA (5 force fields x 2 trial types) showed a main effect of trial type on the amount of extra path (F_(1,8)_ = 19.76, p < 0.01). There was no effect of force field (F_(4,32)_ = .98, p=.43) or interaction effect (F_(4,32)_ = .46, p=.76). Subjects made straighter movements in the vector trials (Figure 9A). For trial duration, a similar ANOVA showed a main effect of trial type (F_(1,8)_ = 10.06, p < 0.02), with an overall effect of force field (F_(4,32)_ = 4.60, p < 0.01), but no differences between force fields in post hoc comparison, and no interaction (F_(4,32)_ = .65, p=.63). Subjects took longer in the vector trials (Figure 9B).

### Discussion

The second experiment extended the results of Experiment 1. We compared the effects that external forces on the hand have on vector reproduction as well as on end-point localization. We found that manual reproduction of both visually presented target positions and movement vectors was not influenced by external forces on the hand. As in experiment 1, individual subjects had different patterns of errors in the end-point trials.

The subjects were aware of the disturbing forces during the experiment (self report) and had the impression that the forces influenced their hand’s path towards the target position. However, in contrast with experiment 1, we did not find a systematic effect of the presence of a force field on the amount of extra path in Experiment 2. This might be because of the alternating tasks. Although the paths in vector trials were very straight, the end-point trials had more extra path than in Experiment 1. The focus on direction reproduction in the vector trials might have changed the way subjects made their movements in the end-point trials. The different set of paths might also have had an influence on the mean amount of extra path.

We argued in the discussion of Experiment 1 that because human arm movements can be seen as vector-based movements followed by end-point control the external forces may not have an influence on movements to end-points, but still have an effect on vector-based movements. The second experiment showed that this was not the case, because increasing the role of vector coding (by having subjects reproduce a visually presented length and direction of a vector) did not increase the influence of external forces. That subjects use a different strategy when reproducing a length in a specified direction than when moving to an indicated endpoint is evident from comparing the extra path and trial duration of the vector trials with those of the end-point trials. Subjects took more time to complete the vector trials, probably because the task is more difficult. They also moved straighter, which is consistent with them reproducing a vector rather than moving towards an (imagined) end-point.

To conclude from Experiment 2, both vector-based movements and movements based on end-points reach the same end-points irrespective of external forces on the hand. This suggests that the external forces did not disturb the proprioception of the hand. In both experiment 1 and 2 the force fields were position-dependent and were irrelevant for the tasks. In a third experiment, dynamic, task-relevant force fields were used to see whether such forces would influence movement end-points. The force fields gave information about the desired movement direction and either assisted or counteracted the subject in making those movements. The assisted movements could be considered as a form of haptic guidance.

## Experiment 3

In the third experiment we repeated the design of the first experiment, but with different force fields for each trial instead of a single force field for a block of trials. In this experiment the force fields gave information about the task, because they were either in the direction of the desired movement (‘assisting’) or in the opposite direction (‘resisting’). We expected the assisting forces to help the subject to make the movement, which could be reflected in less extra path.

### Methods

The subject had to reach the positions of a sequence of targets (in a horizontal plane) with the handle of a force feedback device, like in Experiment 1. Again a force field was imposed on the subject’s hand through the force feedback device.

#### Subjects

Sixteen subjects (all right-handed, 6 men, mean of 31 years of age) volunteered to take part in the experiment. None of the subjects had taken part in Experiment 1 or 2 before they participated in this experiment. All were naive about the purpose of the experiment. All subjects reported (corrected-to-) normal vision.

#### Stimulus and Apparatus

**Figure 10:**
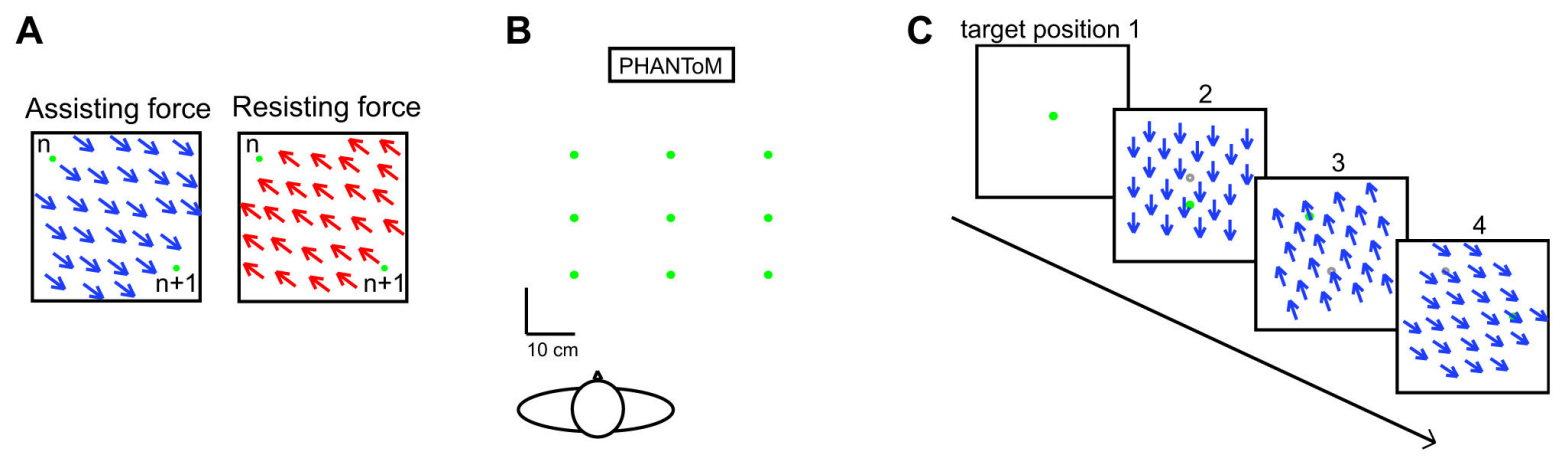
Methods of Experiment 3. A) Top view of the force fields for a movement from position n to position n+1, which in this case is closer and to the right. Blue arrows show the assisting forces, which could be 1N or 2N and red arrows show the resisting forces, which could also be 1N or 2N. B) Top view of the nine target positions relative to the subject. C) Example sequence of stimuli for the first four trials in an assisting force field. First a green target was shown at the origin; the subject moved to that position and pressed the button on the phantom; then a new green target appeared with a force of 1N pulling the hand in the direction of the target; on reaching the target the subject pressed button again, and a new target and force appeared, and so on. The blue arrows indicate the constant force in the direction from the previous (open grey circle) to the current target position.

The apparatus was identical to that in Experiment 1. There were five different force fields that were presented in the workspace: no forces, assisting forces of 1N and 2N, and resisting forces of 1N and 2N (Figure 10A). The force field was applied by the PHANToM at the start of the trial and remained constant during the trial. For the next trial the force instantly changed direction, and was then constant during the trial again. The forces were independent of horizontal or vertical position. The origin of the workspace was about 30 cm in front of the subject’s right shoulder. The visual targets were shown at nine different positions: at the origin, 20 cm left or right of the origin, 14 cm closer to the subject or 12 cm further from the subject than the origin, and at all possible combinations of these deviations from the origin (Figure 10B).

#### Procedure

The procedure of the experiment was identical to that in Experiment 1. An example sequence in an assisting force field is shown in Figure 10C. Again, subjects did not receive any feedback during the experiment other than from their own proprioception.

Each force field was presented to every subject as a block of 74 trials in which each target position was presented 8 times. The order of the trials was semi random as in Experiment 1.

The force fields were presented in a counterbalanced order across subjects. A block of trials (one force field) took the subjects about 4-5 minutes. After each block there was a break of 2-3 minutes to avoid fatigue. On average it took the subjects 40 minutes to complete the whole experiment.

#### Analysis

The means of the movement end-points were calculated for each subject, force field and target position. These means were compared with the positions of the visually presented targets. Furthermore, to measure the amount of extra path, the movement paths were analyzed as in Experiment 1.

### Results

**Figure 11:**
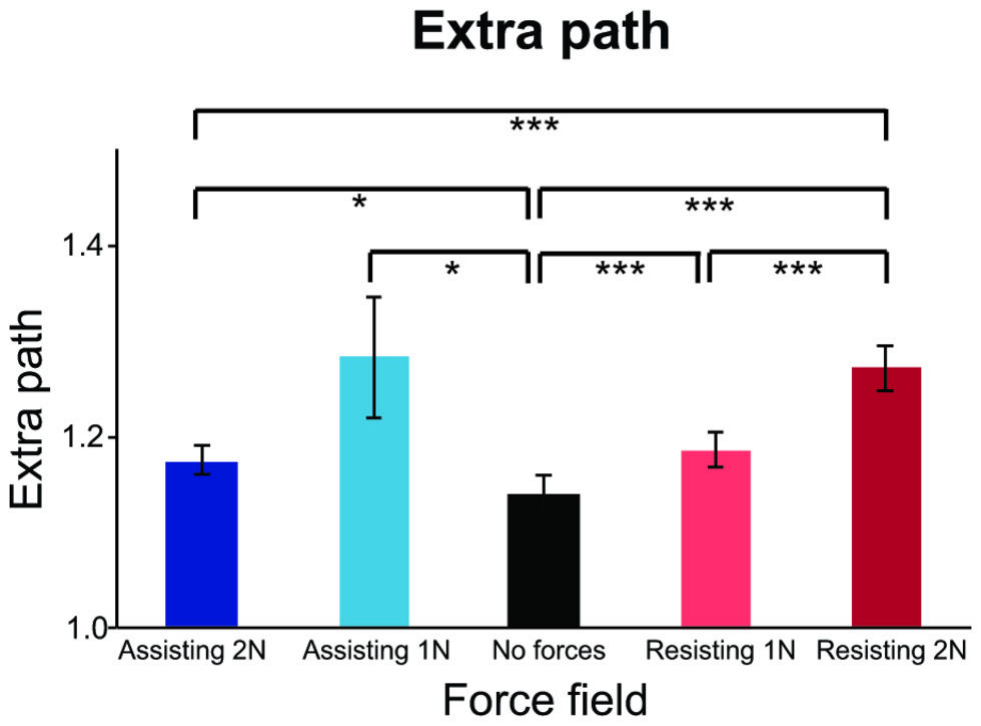
Mean extra path for the different force fields. Each bar shows the mean of the extra path for all subjects in Experiment 3. Error bars show SE’s. The extra path is shorter without forces than with forces. The asterisks denote that the difference is significant (* = p < 0.05, *** = p < 0.001).

Also in experiment 3, no effects of the force fields were found on the end positions, although individual subjects made reproducible errors. Also, as in Experiment 1, the amount of extra path was significantly influenced by the force fields (F_(1.3, 19.6)_ = 5.92, p < 0.02, with Greenhouse-Geisser correction) (Figure 11). Post-hoc comparisons showed that all force fields gave rise to significantly larger values of extra path than without forces (all p < 0.05). There were also differences in extra path between various force fields (Figure 11).

### Discussion

In experiment 3 we showed that external forces that either help or hinder the movements do not influence the reached end-points. The results are comparable with the results of the uninformative external forces in Experiments 1 and 2. As in the previous experiments, the force fields did influence the movement paths. The amount of extra path was significantly higher for movements in the presence of force fields than without forces. Intuitively we did not expect that the amount of extra path was also higher than that without forces for assisting force fields, because these forces guide the movement in the right direction. Additional analyses revealed that the higher amount of extra path was not caused by a larger overshoot in the presence of assisting force fields. Neither were there systematic differences in the maximal deviation perpendicular to the shortest path between movements with and without force fields. Thus, the increase in the amount of extra path with force fields was not easily related to the force fields themselves, but seems to be more complex. Factors that might influence the extra path could be the fact that the force fields might not support the most natural path, and the unpredictability of the force fields (because the direction of the force field changed after every trial, and subjects might need some time to get used to the new direction).

The small difference in the amount of extra path might be relevant for haptic guiding. In modern technology we see that haptic guiding or ‘shared control’ is a promising method to decrease human workload and increase performance (e.g., [25]). Shared control means that technology and humans both contribute to the task. The human is always in final control, but the technology guides the human in the right direction. Shared control could guide steering in a car for lane keeping and curve negotiation [25,26] or guide instruments in tele-operation systems [27]. Shared control could consist of a force that pushes the person’s hand gently in the right direction (as our assisting force fields do). In this study we found that an assisting force influences the subject’s movement paths in a way that increases the path length. A larger extra path, which arises from moving less straight, is a factor that could be undesirable in force-guided systems. Further research is needed to investigate whether the presence of assisting or resisting forces structurally changes the perception of the characteristics of the movement (length, direction etc.) and whether this influences performance. It might also be interesting to see how providing visual feedback affects the interaction between force fields and movement paths.

## General Discussion

In this paper we showed that external forces do not influence proprioceptive localization (within the range of forces and locations tested), which means that the difference between subjects is robust to external forces. This was unexpected based on theoretical considerations and the results of the study of Debats et al [20]. The results are, however, consistent with the results of Cordo & Flanders’ single joint experiment [21]. Although their methods differed considerably from the methods we used, the forces did not change the proprioceptively judged position in both experiments. Furthermore, our results are in line with previous reports that the mismatch between the proprioceptive localization of the arm and visual localization of the target, when asked to align the two, differs across subjects [8–11].

We did find an effect of the forces we used on the movement paths in the end-point trials. Thus forces influence the position of the hand during the movement, but subjects are able to compensate for this (to overcome the forces on the path) with end-point based control. Experiment 2 showed that proprioception was also robust in a vector reproduction task. The last experiment showed that external forces that help rather than hinder the movements do not change the movement end-points either. Again, we found an effect on the movement paths.

People combine afferent and efferent signals into an overall proprioceptive sense of position and movement. During the movement people use all such proprioceptive information to guide their movement, but the final adjustments are presumably made on the basis of the sense of position. In this study we found that the combined position sense is not disturbed by external forces at the end-effector. Although the relationship between position and both the efferent and the afferent information must have changed due to the external forces, the overall position sense is robust. One important difference between efferent and afferent information is their timing. As efferent information leads a movement, and afferent information is delayed with respect to the movement, the two might be in conflict during the movement (e.g. [28]). This conflict in position judgment during a movement might be one of the reasons why the movement paths are disturbed, without effects on the proprioceptive position sense at the endpoints.

To conclude, we found that humans are able to compensate for external horizontal forces applied to the hand both in localization and in reproducing a direction and length.
